# Effectiveness and safety of azvudine in COVID-19: A systematic review and meta-analysis

**DOI:** 10.1371/journal.pone.0298772

**Published:** 2024-06-13

**Authors:** Bahman Amani, Behnam Amani

**Affiliations:** Department of Health Management and Economics, School of Public Health, Tehran University of Medical Sciences, Tehran, Iran; Universitas Pelita Harapan, INDONESIA

## Abstract

**Objective:**

The aim of this study was to assess the effectiveness and safety of azvudine in treating coronavirus disease 2019 (COVID-19) caused by severe acute respiratory syndrome coronavirus 2 (SARS-COV-2).

**Methods:**

A search was carried out in PubMed, Cochrane Library, Web of Science, medRxiv, and Google Scholar until October 20, 2023. The Cochrane risk of bias tools were used to assess the quality of included studies. Comprehensive Meta-Analysis software was used to analyze data.

**Results:**

Twenty-one studies including 10,011 patients were examined. The meta-analysis results showed that azvudine and standard of care/placebo (SOC/PBO) were significantly different concerning mortality rate (risk ratio [RR] = 0.48, 95% confidence interval [CI]: 0.40 to 0.57) and negative polymerase chain reaction (PCR) conversion time (standard mean difference = - 0.75, 95% CI: -1.29 to—0.21). However, the two groups did not show significant differences concerning hospital stay, intensive care unit (ICU) admission, and need for mechanical ventilation (P > 0.05). On the other hand, azvudine and nirmatrelvir-ritonavir were significantly different in mortality rate (RR = 0.73, 95% CI: 0.58 to 0.92), ICU admission (RR = 0.41, 95% CI: 0.21 to 0.78), and need for mechanical ventilation (RR = 0.67, 95% CI: 0.51 to 0.89), but the two treatments were not significantly different in negative PCR conversion time, and hospital stay (P > 0.05). The incidence of adverse events between groups was not significant (P > 0.05). The certainty of evidence was rated as low or moderate.

**Conclusions:**

The antiviral effectiveness of azvudine against SARS-COV-2 is questionable with regard to the certainty of evidence. Further research should be conducted to establish the effectiveness and safety of azvudine in COVID-19.

## Introduction

Current therapeutic strategies have shifted towards vaccination, along with pharmaceutical interventions, for treating coronavirus disease 2019 (COVID-19) caused by severe acute respiratory syndrome coronavirus 2 (SARS-CoV-2) [1]. Research has established the effectiveness of antiviral pharmaceutical agents such as nirmatrelvir-ritonavir, remedsivir, and molnupiravir, in treating patients affected by SARS-CoV-2 variants [2–4]. Furthermore, published studies have indicated that these antiviral agents were more effective in COVID-19 patients already receiving SARS-CoV-2 vaccines [5]. Recently, several studies have evaluated the use of a new oral antiviral agent, azvudine, for COVID-19 patients [6–8]. Azvudine, or 2 -deoxy-2 -β-fluoro-4 –azidocytidine, can be characterized as a nucleoside reverse transcriptase inhibitor and has demonstrated in vitro antiviral activity, alone or in combination with other antiretroviral agents against HIV-1, HIV-2,[9] HBV, and HCV [10]. The application of this antiviral drug in treating adult patients with HIV-1 was approved by China’s National Medical Products Administration (NMPA) in July 2021 [11]. Subsequently, on July 25, 2022, the use of azvudine was recommended and approved by NMPA for treating adult patients with COVID-19 [11]. The treatment mechanism of azvudine as a potential RNA-dependent RNA polymerase inhibitor for COVID-19 has been explained as inhibiting SARS-CoV-2 replication and preserving thymus immune function [12]. Several real-world studies have highlighted the greater effectiveness of azvudine in patients with SARS-COV-2 infection [8,13]. Furthermore, two studies demonstrated a higher effectiveness of azvudine compared to nirmatrelvir-ritonavir in improving clinical outcomes of COVID-19 patients [6,7]. However, Gao et al.[14] showed that nirmatrelvir-ritonavir was associated with a shorter reverse transcriptase polymerase chain reaction (RT-PCR) negative conversion time compared to azvudine in COVID-19 patients. Thus, systematic reviews and meta-analyses are needed to examine the therapeutic potential of azvudine compared to other treatments. The current study seeks to investigate the effectiveness and safety of azvudine in treating patients infected with COVID-19.

## Methods

### Research protocol

This systematic review and meta-analysis followed the protocol registered in the International Prospective Register of Systematic Reviews (PROSPERO) with the code CRD42023449248. The research was conducted utilizing the Preferred Reporting Items for Systematic Reviews and Meta-Analysis (PRISMA) protocol (S1 Table) [15].

### Search strategy and literature screening

Two researchers independently searched PubMed, Cochrane Library, Web of Science, medRxiv, and Google Scholar to identify relevant articles published until October 20, 2023. Additionally, the references of each final article were reviewed for potentially additional relevant papers. No language restrictions were applied to the included studies. The key search terms used in this study were “Coronavirus”, “COVID-19”, “SARS-CoV-2”, “azvudine”, and “FNC”. Further details pertaining to the search strategy for finding relevant evidence for each database are presented in S2 Table.

### Study selection

Studies underwent meta-analysis if they met the following requirements: (1) patients with confirmed COVID-19 by RT-PCR test, (2) azvudine as the intervention, (3) placebo (PBO), standard of care (SOC) or other treatments as comparison, and (4) reported the desired effectiveness and safety outcomes (mortality rate, RT-PCR negative conversion, and adverse events). Research investigating healthy subjects, irrelevant outcomes, case series without a control group, and case reports were excluded from the analysis.

### Risk of bias assessment and quality of the evidence

Two authors independently utilized Cochrane risk of bias tools to assess the bias risk of the included studies. The non-randomized studies and randomized clinical trials (RCTs) were assessed using the risk of bias in nonrandomized studies of interventions (ROBINS-I) [16] and the risk of bias (RoB) tools [17], respectively. These tools assess bias risk in several domains, including selection bias, missing data, reported result, measurement of outcomes, and other potential biases. Furthermore, the grading of recommendations assessment, development and evaluation (GRADE) tool was also utilized to assess the certainty of evidence [18].

### Data extraction

The two authors independently sought to extract the following data: (1) research profile (first author, publication year, place, and research design), (2) patient data (total sample size, sex, mean age, comorbidity percentage, and COVID-19 vaccination status), (3) treatment interventions (dosage, sample size, mean age, and duration of treatment), (4) reported outcomes of interest (mortality rate, negative PCR conversion time, ICU admission, and need for mechanical ventilation), and (5) safety outcomes (any adverse events).

### Statistical analyses

Comprehensive Meta-Analysis (CMA) software, version 3.3 was employed to perform a meta-analysis, comparing the effectiveness and safety of azvudine with SOC/PBO and nirmatrelvir–ritonavir. The analyzed outcomes of interest included mortality rate (death due to any causes), negative PCR conversion time (the mean time to negative RT-PCR conversion), length of hospital stay (the average number of hospitalization from admission to discharge), admission to intensive care unit (ICU), the need for mechanical ventilation, and the incidence of adverse events. The standard mean difference (SMD) and the risk ratio (RR) were used for continuous and dichotomous variables, respectively, both with 95% confidence interval (CI). Statistical heterogeneity was assessed using I^2^>50% or P <0.1 values. The random-effect and fixed-effect models were employed for data with substantial heterogeneity and otherwise, respectively. Subgroup analyses were carried out by age group, study design, sample size, and propensity score matching (PSM). Besides, the robustness of the evidence was examined through a sensitivity analysis. Out of the 21 included studies, four [6,7,19,20] were conducted in the Xiangya hospital between December 5, 2022, and January 13, 2023. As it was likely that patients included in one study were also included in others, we attempted to obtain more information from the authors regarding the data source and potential overlaps in participants. Due to no response from the authors, we hypothesized that the data sources of these studies were identical. Out of the four studies, two [6,7] compared azvudine with nirmatrelvir–ritonavir, and in other two studies, [19,20] compared azvudine with SOC. We decided to exclude studies conducted by Dian et al. [7] and Shen et al. [19] in which the control treatments were nirmatrelvir-ritonavir and SOC, respectively, and had smaller sample sizes compared to other two studies. Furthermore, we conducted a sensitivity analysis by excluding studies with potential risk of bias.

## Results

### The process of the study selection

**Fig 1:**
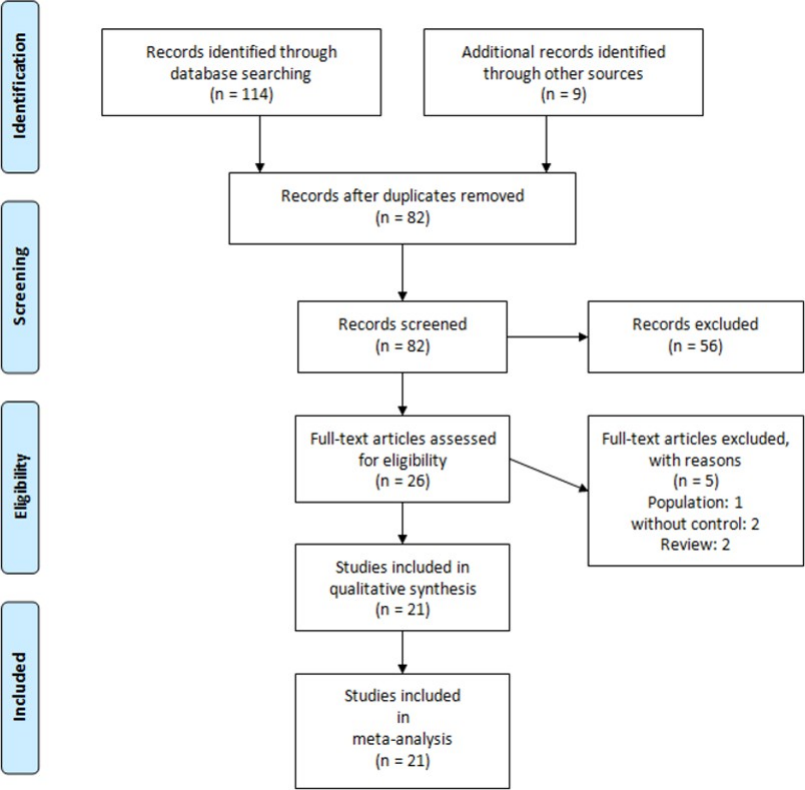
PRISMA flow chart of literature screening.

Fig 1 illustrates the selection and screening process based on titles, abstracts, and full texts of the studies. The initial search identified 123 articles. After removing duplicates and screening by title and abstract, 26 eligible studies remained for full-text investigation. Five studies were excluded due to the lack of a control group and other reasons [12,21–24]. Ultimately, meta-analyses were conducted on 21 articles, [6–8,13,14,19,20,25–38] including 18 retrospective cohort studies and three RCTs involving 10,011 patients. All included studies, except two [26,27], were conducted in China and during the SARS-CoV-2 Omicron variant wave. Patients received azuduvine 5 mg orally once daily for 5 days. Nirmatrelvir–ritonavir was administrated as 100/300mg every 12 h for 5 days. Most retrospective studies used PSM. Studies used SOC, PBO, and nirmatrelvir-ritonavir as control groups. Table 1 presents the main features of the included articles.

**Table 1:** Characteristics of included studies in the meta-analysis.

First author	Year	Country	Design	Sample size	Male %	Severity of COVID-19	Azvudine				Comparison				
							N	Mean age	Comorbidity (%) ^a^	COVID‐19 vaccination ^b^	Control(s)	N	Mean age	Comorbidity (%)	COVID‐19 vaccination
Chen [13]	2023	China	RCS	207	57	MM	166	36	19.9	NR	SOC	41	29	12.2	NR
Deng [6]	2023	China	RCS	562	60	MS	281	67.5	83.3	49.1	NMV/r	281	67.4	84.3	49.1
Dian [7]	2023	China	RCS	456	64	MS	228	69.21	100	48.2	NMV/r	228	70.44	100	48.2
Fu [34]	2023	China	RCS	140	64	MC	62	45.0	69.4	NR	NMV/r, NMV/r +AZ	78	NR	59.2	NR
Gao [14]	2023	China	RCS	134	60	NR	67	70.5	NR	NR	NMV/r	67	70.2	NR	NR
Han [28]	2023	China	RCS	856	NR	NR	428	62.7	43.5	NR	SOC	428	61.1	38.3	NR
Liu [29]	2023	China	RCS	642	89	MC	206	67.8	48	NR	SOC	436	70.0	47.1	NR
Qi [30]	2023	China	RCS	13	69	MC	6	65.5	NR	NR	SOC	7	72.3	NR	NR
Qinqin Zhao [35]	2023	China	RCS	286	61	MS	143	76.4	34.2	NR	N/R	143	76.8	35.6	NR
Ren [8]	2020	China	RCT	20	60	MM	10	52	10	NR	SOC	10	50.5	10	NR
Shang [31]	2023	China	RCS	364	50	NR	182	54	9.8	54 2dose	SOC	182	55	11.5	50
Shao [32]	2023	China	RCS	966	61	SC	177	78.0**	NR	34.7	SOC, NMV/r	789	78.0	NR	34.7
Shen [19]	2023	China	RCS	452	58	MS	226	64.4	41	NR	SOC	226	65.3	50	NR
Silva [26]	2023	Brazil	RCT	281	39.5	Mild	143	NR	NR	89	PBO	138	NR	NR	88
Souza [27]	2023	Brazil	RCT	179	58	Moderate	91	51	NR	3.3	PBO	88	48	NR	3.4
Sun [20]	2023	China	RCS	490	63	MS	245	69.1	100	48.2	SOC	245	69.2	100	49.0
Wei [25]	2023	China	RCS	725	64	MC	461	68	24	NR	NMV/r	264	65	25	NR
Xiang Zhao [36]	2023	China	RCS	227	46	MC	82	51	28.1	78	NMV/r	145	46.5	19.3	81.4
Yang [33]	2023	China	RCS	804	55	MM	317	67	51.4	63.4	SOC	487	61	56.2	81.1
Yiling Zhou [37]	2023	China	RCS	1154	61	MS	311	70	35.7	NR	SOC, NMV/r	843	69, 73	47.3, 35.7	NR
Zong [38]	2023	China	RCS	585	56	MC ^c^	195	67.8	45	NR	SOC	390	68.2	37	NR

AZ, azvudine; MC, mild to critical; MM, mild to moderate; MS, mild to severe, N, number; NR, not reported; NMV/r, nirmatrelvir/ritonavir; PBO, placebo; RCT, randomized clinical trial, RCS, retrospective cohort study; SOC, standard of care.

a Having at least one comorbidity.

b Receipt of ≥1 dos e SARS-CoV-2 vaccine.

c Moderate to critical.

### Assessing risk of bias and quality of the evidence

Out of the three RCTs, two had a low risk of bias. However, the risk of bias of Ren’s study [8] was moderate due to of lack of patient blinding (S3 Table). The retrospective cohort studies had acceptable quality according to the ROBINS-I tool (S4 Table). Additionally, the quality of the evidence for each outcome is presented in S5 Table, with the certainty of evidence rated as low or moderate.

### Primary effectiveness outcomes

#### Mortality rate

**Fig 2:**
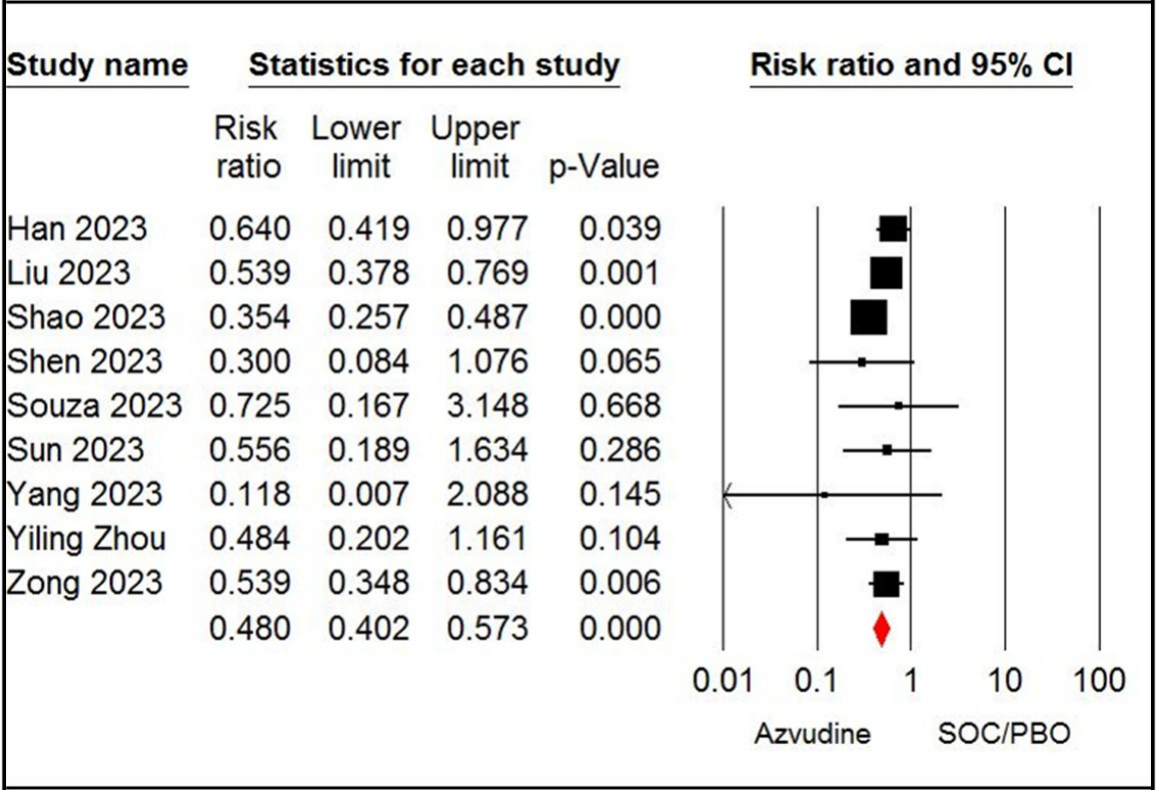
Forest plot of Azvudine vs. standard of care/placebo (SOC/PBO) for mortality rate.

**Fig 3:**
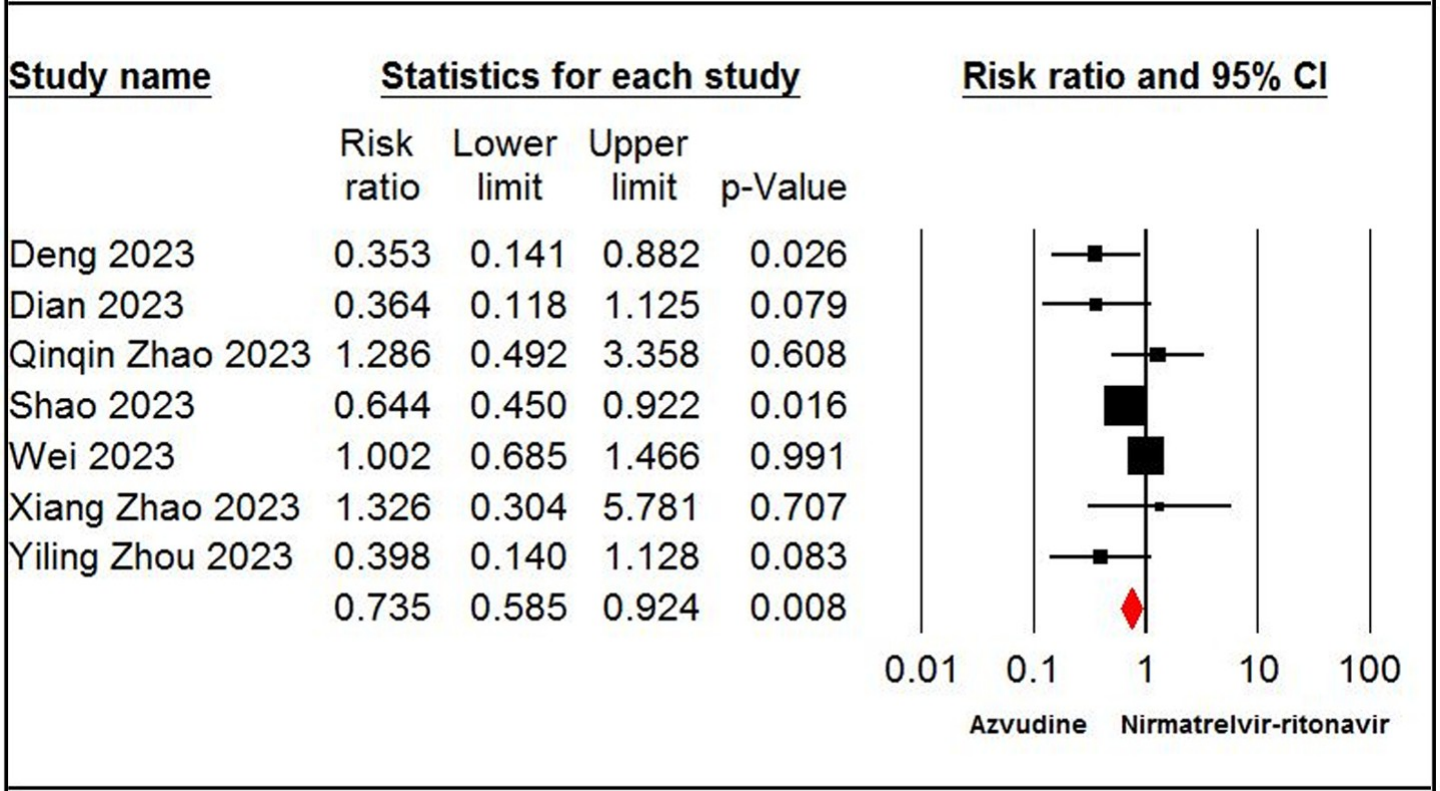
Forest plot of Azvudine vs. nirmatrelvir-ritonavir for mortality rate.

The pooled estimate of nine studies [19,20,27–29,32,33,37,38] showed a significant difference in mortality rate between patients receiving azvudine and SOC (RR = 0.48, 95% CI: 0.40 to 0.57, P < 0.001, I^2^ = 0%, GRADE certainty: moderate) (Fig 2). According to the pooled estimate, seven studies [6,7,25,32,35–37] revealed a significant difference in mortality rate between the azvudine and nirmatrelvir-ritonavir groups (RR = 0.73, 95% CI: 0.58 to 0.92, P < 0.05, I^2^ = 41%, GRADE certainty: moderate) (Fig 3).

#### Negative PCR conversion time

**Fig 4:**
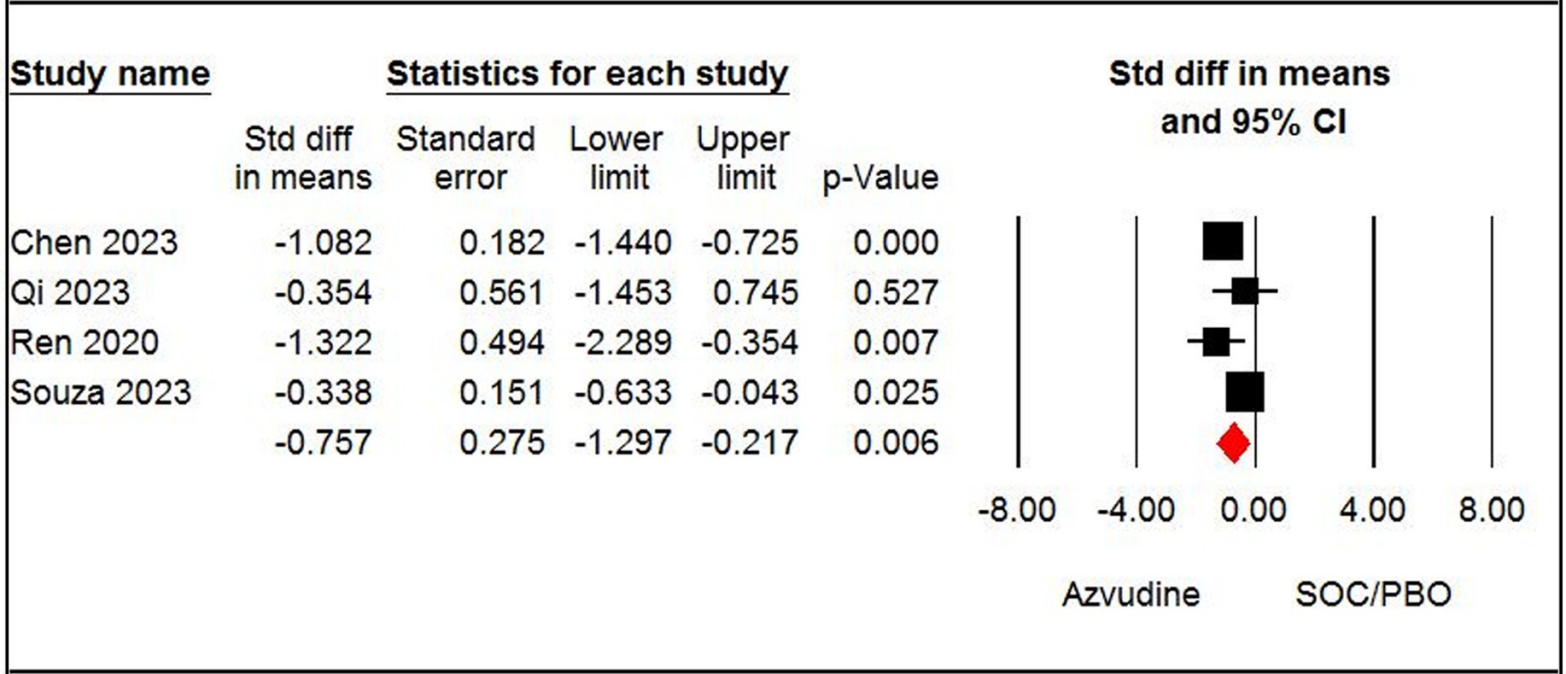
Forest plot of Azvudine vs. standard of care/placebo (SOC/PBO) for negative PCR conversion time.

**Fig 5:**
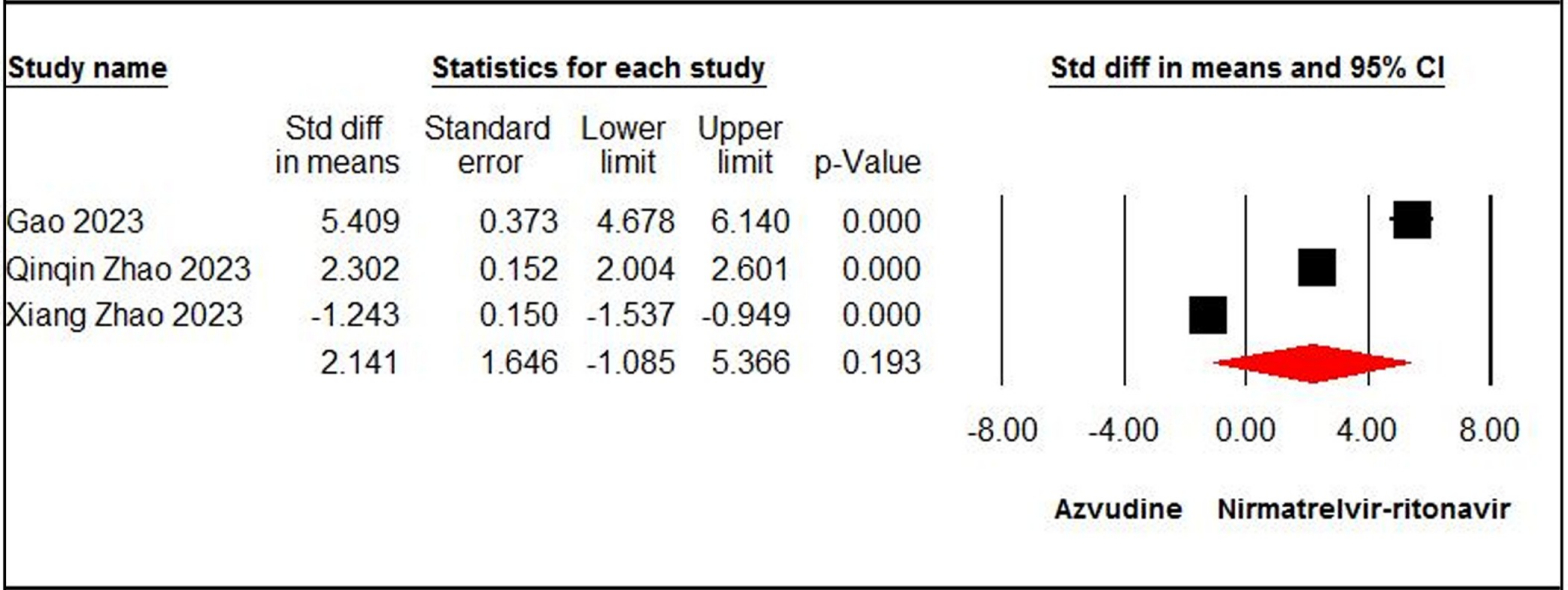
Forest plot of Azvudine vs. nirmatrelvir-ritonavir for negative PCR conversion time.

The pooled estimate of four papers [8,13,27,30], showed a significant difference in negative PCR conversion time between the azvudine and SOC/PBO groups (SMD = - 0.75, 95% CI: -1.29, - 0.21; P < 0.05, I^2^ = 75%, GRADE certainty: low) (Fig 4). The pooled estimate of three papers [14,35,36] highlighted no significant differences between the azvudine and nirmatrelvir-ritonavir groups concerning negative PCR conversion time (SMD = 2.14, 95% CI: -1.08, 5.36; P = 0.19, I^2^ = 99%, GRADE certainty: low) (Fig 5).

#### Length of hospital stay

**Fig 6:**
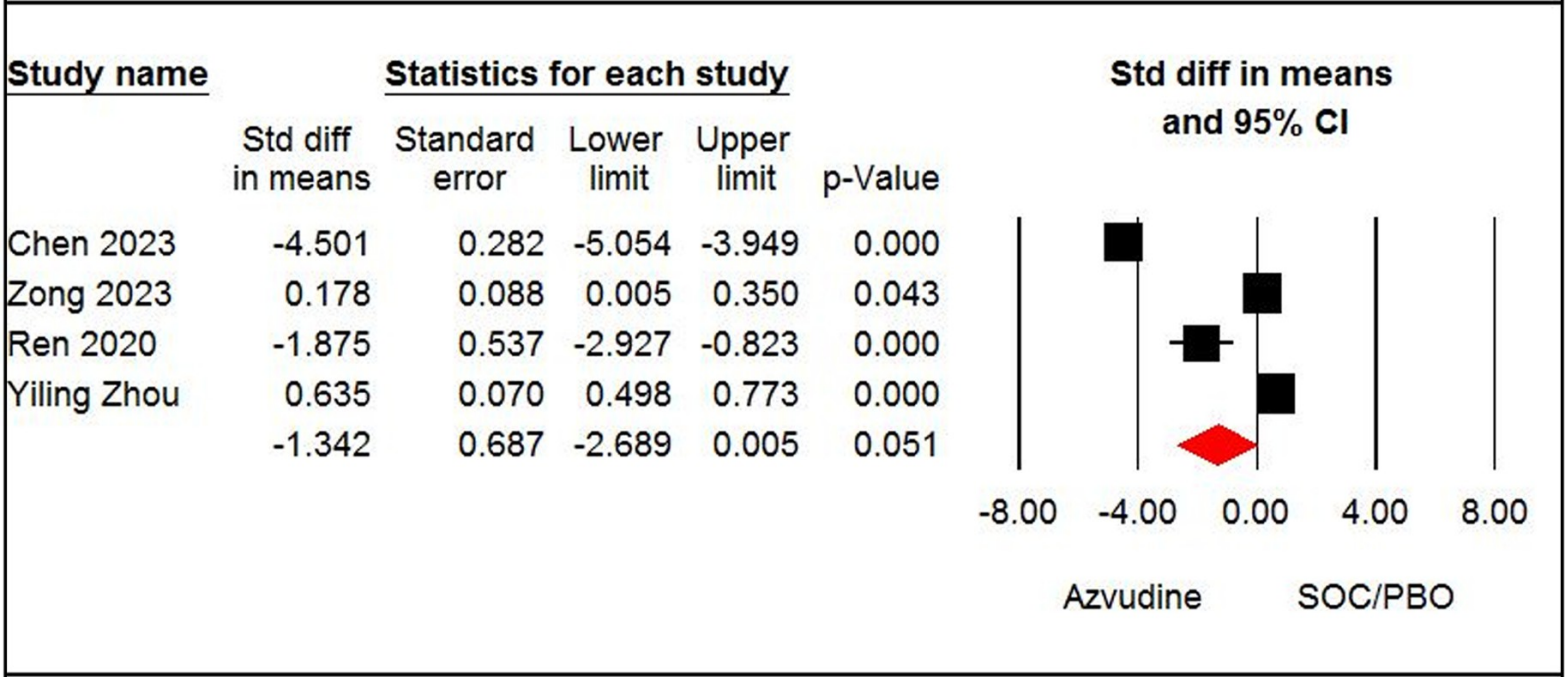
Forest plot of Azvudine vs. standard of care/placebo (SOC/PBO) for hospital stay.

**Fig 7:**
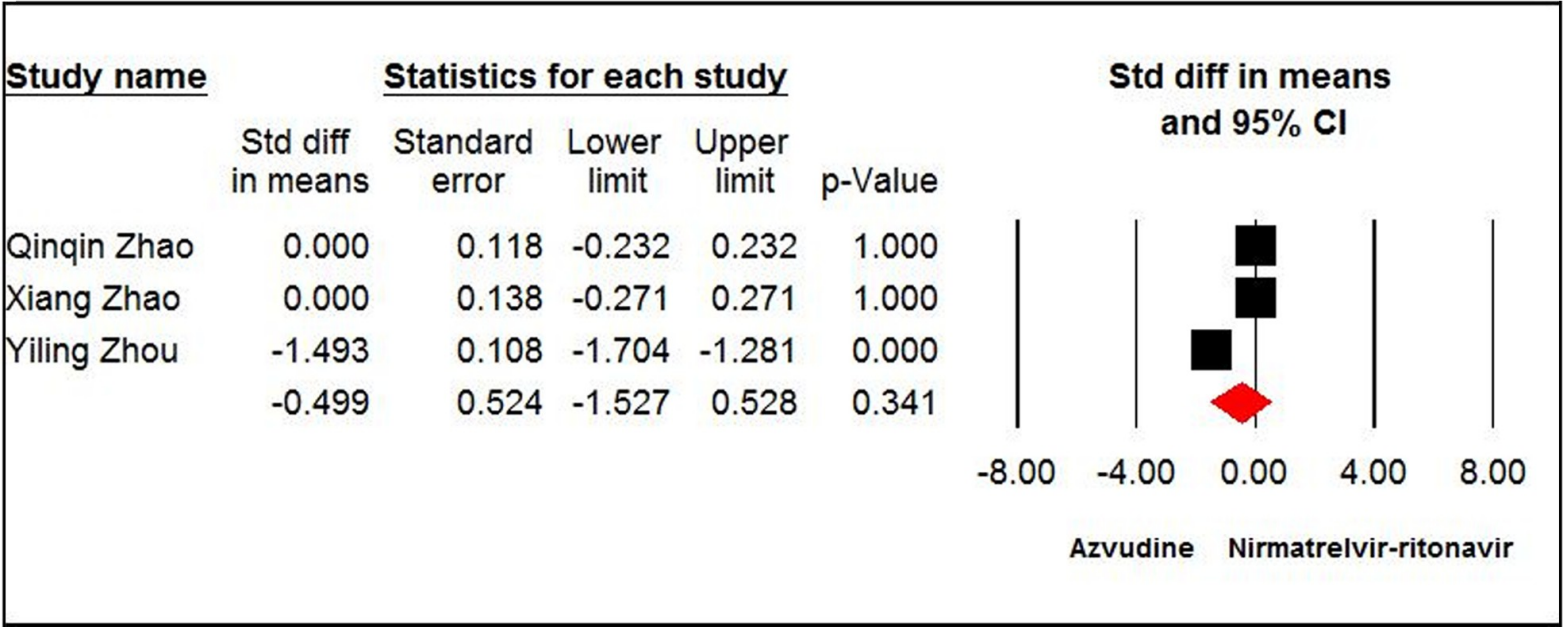
Forest plot of Azvudine vs. nirmatrelvir-ritonavir for hospital stay.

The pooled estimates of four papers [8,13,37,38] with 1801 patients indicated that the azvudine and SOC/PBO groups were not significantly different in length of hospital stay (SMD = - 1.34, 95% CI: -2.68, 0.005; P = 0.05, I^2^ = 99%, GRADE certainty: low) (Fig 6). According to the pooled estimate of three papers, [35–37] the azvudine and nirmatrelvir-ritonavir groups were not significantly different regarding length of hospital stay (SMD = - 0.49, 95% CI: -1.52, 0.52; P = 0.34, I^2^ = 98%, GRADE certainty: low) (Fig 7).

### Secondary effectiveness outcomes

#### ICU admission

As shown by the pooled estimate of three studies [19,20,27] with 1121 patients, those receiving azvudine were not significantly different from patients receiving SOC/PBO in terms of ICU admission (RR = 0.68, 95% CI: 0.23 to 2.03, P = 0.49, I^2^ = 0%, GRADE certainty: moderate) (S1 Fig). The pooled estimate of four studies [6,7,25,34] showed a significant difference between the azvudine and nirmatrelvir-ritonavir groups concerning ICU admission (RR = 0.41, 95% CI: 0.21 to 0.78, P < 0.05, I^2^ = 0%, GRADE certainty: moderate) (S2 Fig).

#### Need for mechanical ventilation

Four studies [19,20,37,38] involving 2516 patients reported the need for mechanical ventilation in patients taking azvudine and SOC. The pooled estimate of the included studies revealed that the two groups were not significantly different concerning the need for mechanical ventilation (RR = 0.90, 95% CI: 0.54 to 1.50, P = 0.69, I^2^ = 19%, GRADE certainty: moderate) (S3 Fig). Based on the pooled estimate of the included papers, the azvudine and nirmatrelvir-ritonavir groups were significantly different regarding the need for mechanical ventilation (RR = 0.67, 95% CI: 0.51 to 0.89, P < 0.05, I^2^ = 0%, GRADE certainty: moderate) (S4 Fig).

### Safety outcomes

#### Adverse events

**Fig 8:**
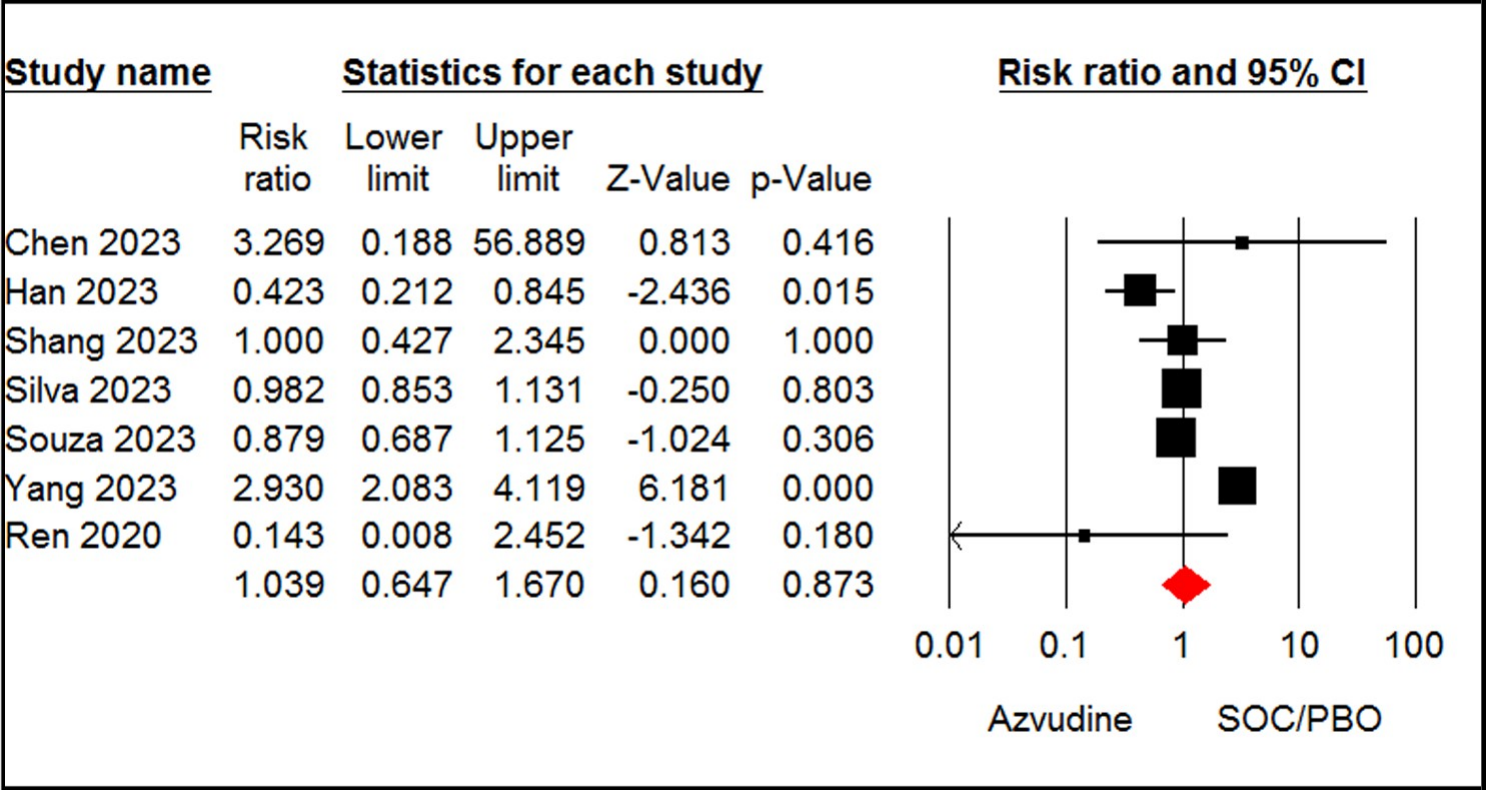
Forest plot of Azvudine vs. standard of care/placebo (SOC/PBO) for adverse events.

Seven studies [8,13,26–28,31,33] involving 2742 patients reported the incidence of adverse events in patients taking azvudine and SOC/PBO. The pooled estimate of the included studies revealed no significant difference between the two groups in terms of adverse events (RR = 1.03, 95% CI: 0.64 to 1.67, P = 0.87, I^2^ = 87%, GRADE certainty: low) (Fig 8). According to the pooled estimate of three papers [25,34,35], the azvudine and nirmatrelvir-ritonavir groups were not significantly different regarding incidence of adverse events (RR = 0.60, 95% CI: 0.32 to 1.13, P = 0.11, I^2^ = 50%, GRADE certainty: low) (S5 Fig).

### Subgroup and sensitivity analyses

Table 2 presents the subgroup analysis results based on age group, study design, sample size, and PSM. As shown by the sensitivity analysis, the outcomes of mortality rate, composite disease progression outcome, ICU admission, and need for mechanical ventilation did not change significantly (Table 2). Moreover, no significant change was observed for outcomes of mortality rate and hospitalization when excluding articles with high risk of bias.

**Table 2:** Subgroup and sensitivity analyses for effectiveness outcomes.

Analysis	No. of studies	Sample size	Point estimate (95% CI)	P-value	Heterogeneity		
					Q-value	P-value	I-squared
Sensitivity analysis							
Mortality rate, AZ vs. SOC/PBO (excluding Shen et al.)	8	6186	0.48 [0.40, 0.57]	<0.001	7.24	0.40	3.31
Mortality rate, AZ vs. NMV/r (excluding Dian et al.)	6	2733	0.75 [0.60, 0.95]	0.020	8.72	0.12	42.66
ICU admission, AZ vs. SOC/PBO (excluding Shen et al.)	2	669	0.95 [0.27, 3.34]	0.94	0.50	0.47	0.00
ICU admission, AZ vs. NMV/r (excluding Dian et al.)	3	1398	0.39 [0.20, 0.75]	0.005	1.17	0.55	0.00
MV, AZ vs. SOC/PBO (excluding Shen et al.)	3	2064	0.97 [0.57, 1.63]	0.91	1.20	0.54	0.00
MV, AZ vs. NMV/r (excluding Dian et al.)	4	1874	0.69 [0.52, 0.91]	0.01	2.67	0.44	0.00
Hospital stay, AZ vs. SOC/PBO (excluding Ren et al.)	3	1781	- 1.18 [-2.70, 0.33]	0.12	315.24	<0.001	99.36
Negative PCR conversion time (excluding Ren et al.)	3	399	- 0.63 [-1.23, -0.03]	0.03	10.15	0.006	80.31
Subgroup analysis							
Mortality rate by age group (AZ vs. SOC/PBO)							
≥ 60	8	6459	0.47 [0.39, 0.57]	<0.001	7.46	0.38	6.21
< 60	1	179	0.72 [0.16, 3.14]	0.66	0.00	1.00	0.00
Mortality rate by study design (AZ vs. SOC/PBO)							
RCS	8	6459	0.47 [0.39, 0.57]	<0.001	7.46	0.38	6.21
RCT	1	179	0.72 [0.16, 3.14]	0.66	0.00	1.00	0.00
Mortality rate by sample size (AZ vs. SOC/PBO)							
< 500	3	1121	0.48 [0.23, 1.00]	0.05	0.89	0.64	0.00
≥ 500	6	5517	0.48 [0.40, 0.57]	<0.001	6.87	0.23	27.29
Mortality rate by age group (AZ vs. NMV/r)							
≥ 60	5	2676	1.29 [0.58, 2.90]	0.52	0.001	0.97	0.00
< 60	2	513	0.69 [0.55, 0.88]	0.003	8.18	0.08	51.15
Mortality rate by sample size (AZ vs. NMV/r)							
< 500	5	1902	0.65 [0.48, 0.88]	0.006	4.70	0.31	15.03
≥ 500	2	1287	0.85 [0.60, 1.22]	0.39	4.25	0.03	76.48
Mortality rate by PSM (AZ vs. NMV/r)							
Without PSM	2	933	0.61 [0.43, 0.85]	0.005	0.73	0.31	0.00
With PSM	5	2256	0.85 [0.62, 1.16]	0.32	7.49	0.11	46.59
Negative PCR conversion time (AZ vs. SOC/PBO)							
RCS	2	220	- 1.01 [-1.35, -0.67]	<0.001	1.52	0.21	34.40
RCT	2	199	- 0.42 [-0.70, -0.13]	0.003	3.63	0.05	72.47
Hospital stay (AZ vs. SOC/PBO)							
RCS	3	1781	0.27 [0.17, 0.38]	<0.001	315.24	<0.001	99.36
RCT	1	20	- 1.87 [-2.97, -0.82]	<0.001	0.00	1.00	0.00

AZ, azvudine; CI, confidence interval; MV, mechanical ventilation; NMV/r, nirmatrelvir/ritonavir; PBO, placebo; PCR, polymerase chain reaction; RCT, randomized clinical trial, RCS, retrospective cohort study; SOC, standard of care.

## Discussion

The management of the COVID-19 pandemic has become increasingly challenging due to the emergence of several SARS-CoV-2 variants that demonstrate increased transmissibility and resistance to vaccines and treatments [39]. Thus, the development and evaluating of new treatments is of high importance for this condition. Recent research has suggested azvudine as a promising antiviral agent for treating COVID-19 patients [6–8]. The present systematic review and meta-analysis aimed to investigate the available evidence for the effectiveness and safety of azvudine in treating COVID-19 patients.

The meta-analysis results revealed that the use of azvudine in COVID-19 patients was associated with lower death rates compared to SOC/PBO or nirmatrelvir–ritonavir. However, it should be noted that while retrospective studies have shown that exposure to azvudine is associated with lower mortality rate; the antiviral effectiveness of azvudine against SARS-CoV-2 is questionable. The demonstrated effectiveness in real-world studies and retrospective studies may be due to potential bias. The effectiveness of azvudine in reducing COVID-19-related deaths has been the subject of conflicting evidence. While some real-world studies and RCTs have shown a lower risk of death in patients receiving azvudine compared to those receiving SOC/PBO [27] or nirmatrelvir–ritonavir [6,32], other studies [6,7,25,35] found no significant differences between azvudine and nirmatrelvir-ritonavir treatments in reducing mortality rates in COVID-19 patients. Conversely, strong evidence supports the effectiveness of nirmatrelvir-ritonavir in reducing the mortality rate related to COVID-19 [2,40,41]. Meta-analyses of real-world studies on confirmed COVID-19 cases have shown that the administration of nirmatrelvir-ritonavir led to significantly lower rates of death compared to untreated individuals with nirmatrelvir-ritonavir [2,42].

The meta-analysis revealed that compared to SOC/PBO, azvudine was significantly associated with shorter negative PCR conversion time in COVID-19 patients. However, the mean time to negative PCR was statistically similar between patients who received azvudine and those who received nirmatrelvir–ritonavir. Studies have shown the effectiveness of azvudine in reducing negative PCR conversion time. In a randomized, single-arm clinical trial on 31 COVID-19 patients, azvudine was found to effectively treat all patients, achieving 100% viral clearance within 3.29 ± 2.22 days.[12] Additionally, in Chen’s study, the viral clearance rate of patients treated with azvudine and SOC was 100%. However, Gao et al. [14] found a shorter negative PCR conversion time in patients under nirmatrelvir-ritonavir treatment compared to those receiving azvudine. Xiang Zhao et al. [36] also found that nirmatrelvir-ritonavir was associated with shorter SARS-Cov-2 negative conversion in patients suffering from mild COVID-19 (P = 02). However, they found no statistical difference between the two treatments in patients having moderate, severe, and critical COVID-19 (P > 0.05). While the present result showed a slightly better effectiveness of azvudine compared to SOC/PBO in terms of SARS-Cov-2 negative conversion time, current evidence has supported the effectiveness of nirmatrelvir-ritonavir in improving negative PCR conversion time. A meta-analysis of real-world evidence showed that nirmatrelvir- ritonavir may shorten negative PCR conversion time in patients suffering from the SARS-COV-2 Omicron variant [2]. It is important to note that the timing of antiviral agent administration is crucial according to retrospective cohort studies that use PSM. According to Gao et al [23], antiviral agents should be received within 5 days of COVID-19 symptoms onset in order to be more effective in patients. However, since most patients after PSM do not receive these treatment interventions within 5 days of symptom onset, they may not benefit from these interventions. For instance, in study conducted by Deng et al [6], only 9.4% and 18.2% hospitalized patients with COVID‐19 received azvudine and nirmatrelvir-ritonavir within 5 days of symptom onset, respectively.

Based on the meta-analysis, the mean length of hospital stay did not differ between patients receiving azvudine and those treated with SOC/PBO. Multiple real-world studies also found no statistically significant difference in hospital stay length between patients treated with azvudine and SOC [13,38]. However, Ren et al. [8] demonstrated that the mean hospital stay was statistically shorter in the azvudine group compared to the nirmatrelvir-ritonavir group for COVID-19 patients. The present meta-analysis also indicated no statistical difference in hospital stay length between COVID-19 patients in the azvudine and nirmatrelvir-ritonavir groups. Zhao et al. [36] reported that nirmatrelvir- ritonavir appeared to be more effective in reducing hospital stay in patients with mild COVID-19, although this difference was insignificant in patients with moderate, severe, and critical COVID-19.

The results indicated no significant benefits in favor of azvudine compared to SOC/PBO in reducing ICU admission or the need for mechanical ventilation in COVID-19 patients. However, the meta-analysis revealed that azvudine was significantly associated with reduced ICU and need for mechanical ventilation in COVID-19 patients compared to nirmatrelvir–ritonavir. Some real-world studies have indicated a lower percentage of COVID-19 patients needing mechanical ventilation when treated with azvudine compared to nirmatrelvir–ritonavir. Conversely, Deng et al. [6] found that treating hospitalized COVID‐19 patients with azvudine was not associated with improvements in ICU admission, mechanical ventilation, and high‐flow oxygen therapy compared to nirmatrelvir–ritonavir. Nonetheless, a pooled analysis of studies has demonstrated the effectiveness of nirmatrelvir–ritonavir in reducing ICU admission [43].

Regarding safety outcomes, the incidence of adverse events in COVID-19 patients appeared similar between azvudine and SOC/PBO, as well as between azvudine and nirmatrelvir–ritonavir. A meta-analysis by Chen and Tian [22] demonstrated that the incidence of adverse events was significantly lower in patients taking azvudine compared to those receiving SOC/PBO. They reported no significant differences between the Azvudine and SOC/PBO groups concerning the incidence of severe adverse events. Ren et al. [8] reported no adverse events in the azvudine group, whereas 30% of the control patients experienced adverse events such as anorexia, nausea, vomiting, and abdominal pain. However, in Chen’s study,[13] all adverse events occurred among patients receiving azvudine. According to their study, 3.6% of patients treated with azvudine experienced nausea, diarrhea, vomiting, and stomachache. Nonetheless, some studies reported cases of renal injuries in COVID-19 patients treated with azvudine [28,33].

The current research has several limitations worthy of consideration. First, most of the articles investigated in the present meta-analysis were retrospective, which could introduce confounding variables. However, it is important to note that most of these studies used PSM to mitigate these potential confounders. Second, the data source for four studies included in the meta-analysis was obtained from a single hospital during the same time period, which may introduce bias into the results. To address this concern, a sensitivity analysis was conducted by excluding these studies from the analysis. Third, some retrospective studies did not report the specific supportive treatments received by patients in the control groups. As a result, these treatments were considered as SOC in our meta-analysis, potentially introducing bias into the results. Finally, due to incomplete reporting, not all studies included in the analysis reported the prevalence of comorbidities or COVID-19 vaccination status in COVID-19 patients. Consequently, the present findings may not accurately estimate the treatment effect of azvudine in COVID-19 patients.

## Conclusion

The current systematic review and meta-analysis suggest that treatment with azvudine in COVID-19 patients is associated with a reduction in mortality rate and negative PCR conversion time compared to SOC/PBO. However, it does not appear to have a significant effect in reducing hospital stay, ICU admission, and the need for mechanical ventilation. When compared to nirmatrelvir–ritonavir, azvudine showed better effectiveness in improving mortality rate, ICU admission, and the need for mechanical ventilation, but did not demonstrate a clinical benefit in terms of negative PCR conversion time and hospital stay. Nevertheless, the level of certainty regarding the evidence supporting the antiviral effectiveness of azvudine against SARS-CoV-2 is either low or moderate. As such, the current findings are unable to confirm the effectiveness of azvudine in treating COVID-19. In terms of safety outcomes, treating COVID-19 patients with azvudine showed similar safety profiles to SOC/PBO and nirmatrelvir–ritonavir. However, further long-term follow-up is necessary to confirm its safety. These findings may offer valuable insights into the treatment effect of azvudine in patients with COVID-19 for healthcare providers and researchers. Nevertheless, additional research is essential to establish the effectiveness and safety of azvudine in COVID-19 patients.

## Supporting information

S1 Fig Forest plot of azvudine vs. standard of care/placebo (SOC/PBO) for ICU admission. (TIF)

S2 Fig Forest plot of azvudine vs. nirmatrelvir-ritonavir for ICU admission. (TIF)

S3 Fig Forest plot of azvudine vs. standard of care/placebo (SOC/PBO) for need for mechanical ventilation. (TIF)

S4 Fig Forest plot of azvudine vs. nirmatrelvir-ritonavir for Need for mechanical ventilation. (TIF)

S5 Fig Forest plot of azvudine vs. nirmatrelvir-ritonavir for adverse events. (TIF)

S1 Table PRISMA checklist. (DOCX)

S2 Table Search strategy. (DOCX)

S3 Table Risk of bias of included randomized controlled trial (RCT). (DOCX)

S4 Table ROBINS-I tool results for non-randomized studies. (DOCX)

S5 Table Assessment of certainty of evidence using the GRADE approach for included outcomes. (DOCX)
