# Supplementary figures and images for: Effectiveness and safety of azvudine in COVID-19: A systematic review and meta-analysis
Source: PLoS One. 2024 Jun 13;19(6):e0298772. doi: 10.1371/journal.pone.0298772 (PMC11175417; doi:10.1371/journal.pone.0298772)

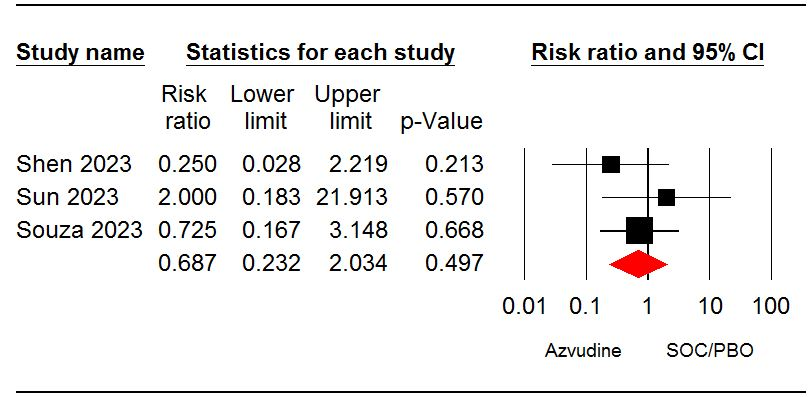

Supplement: S1 Fig — (TIF) [file pone.0298772.s001.tif]

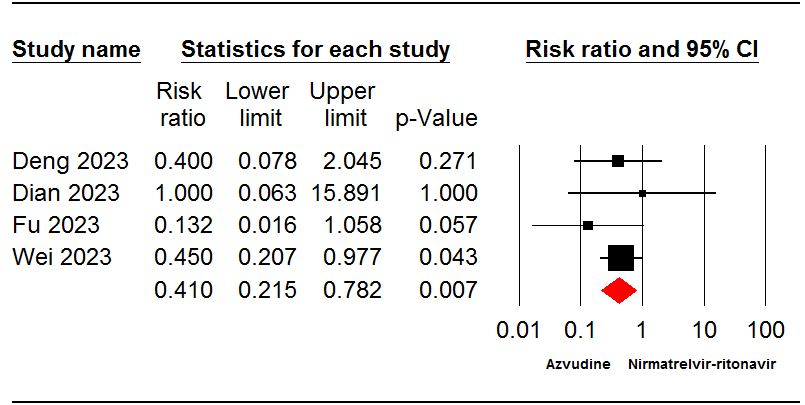

Supplement: S2 Fig — (TIF) [file pone.0298772.s002.tif]

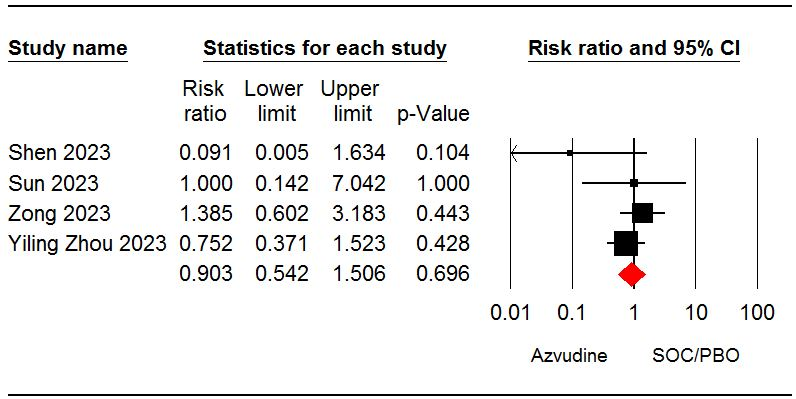

Supplement: S3 Fig — (TIF) [file pone.0298772.s003.tif]

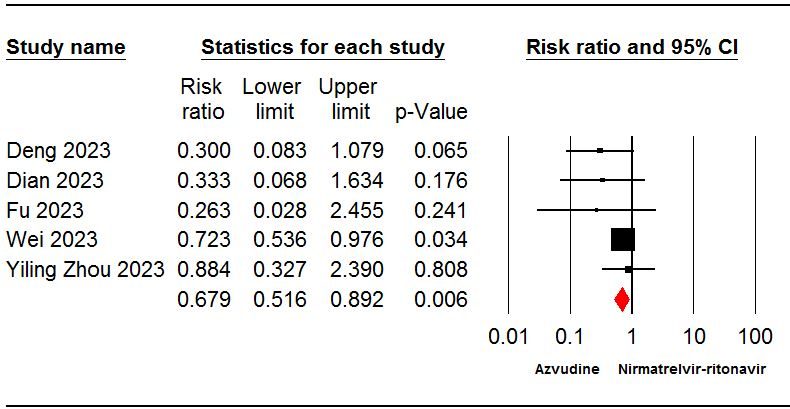

Supplement: S4 Fig — (TIF) [file pone.0298772.s004.tif]

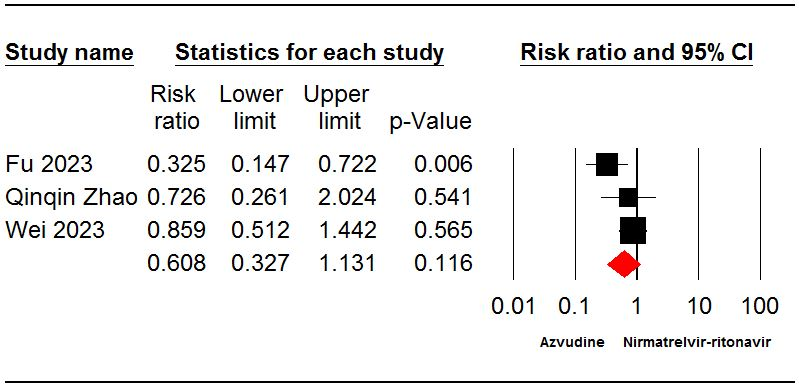

Supplement: S5 Fig — (TIF) [file pone.0298772.s005.tif]
