# Supplementary material for: Effectiveness and safety of azvudine in COVID-19: A systematic review and meta-analysis
Source: PLoS One. 2024 Jun 13;19(6):e0298772. doi: 10.1371/journal.pone.0298772 (PMC11175417; doi:10.1371/journal.pone.0298772)
Supplement: S2 Table — (DOCX) [file pone.0298772.s007.docx]

**S1 Table 1 - Search strategy**

| **Cochrane Library** |
| --- |
| MeSH descriptor: [Coronavirus] explode all trees OR MeSH descriptor: [SARS-CoV-2] explode all trees OR ("coronavirus"):ti,ab,kw OR (COVID-19):ti,ab,kw OR ("coronavirus infection"):ti,ab,kw OR (2019 nCoV):ti,ab,kw OR (2019nCoV):ti,ab,kw OR (nCov 2019):ti,ab,kw OR (SARS CoV2):ti,ab,kw OR (SARS CoV 2):ti,ab,kw OR (SARSCoV2):ti,ab,kw OR (SARSCoV 2):ti,ab,kw OR (severe acute respiratory syndrome coronavirus 2):ti,ab,kw AND "Azvudine" OR "FNC" OR "2′-deoxy-2′-β-fluoro-4′-azidocytidine" OR " RO-0622" OR " RO 0622" OR " RO0622" |
| **PubMed** |
| **#1** (COVID‐19[MeSH Terms]) OR (Coronavirus[MeSH Terms])) OR (SARS‐CoV‐2[MeSH Terms])) OR (coronavirus[Title/Abstract])) OR (COVID-19[Title/Abstract])) OR (coronavirus infection[Title/Abstract])) OR (2019 nCoV[Title/Abstract])) OR (2019nCoV[Title/Abstract])) OR (nCov 2019[Title/Abstract])) OR (SARS CoV2[Title/Abstract])) OR (SARS CoV 2[Title/Abstract])) OR (SARSCoV2[Title/Abstract])) OR (SARSCoV 2[Title/Abstract])) OR (severe acute respiratory syndrome coronavirus 2[Title/Abstract])) OR (novel corona virus disease[Title/Abstract])) OR (corona virus disease 2019[Title/Abstract])) OR (coronavirus disease 2019[Title/Abstract])) OR (novel coronavirus pneumonia[Title/Abstract])) OR (novel corona virus pneumonia[Title/Abstract])  **#2** (Azvudine OR FNC OR 2′-deoxy-2′-β-fluoro-4′-azidocytidine OR RO-0622 OR RO 0622 OR RO0622)  **#3** #1 AND #2 |
| **Web of Science** |
| **1.** TI**= (**Coronavirus OR "COVID-19" OR "COVID OR COVID19" OR "SARS‐CoV2" OR "SARS-CoV-2" OR SARSCoV2 OR "SARSCoV‐2" OR "SARS coronavirus 2" OR "2019 nCoV" OR "2019nCoV" OR "2019‐novel CoV" OR "nCov 2019" OR "nCov 19" OR "coronavirus infection" OR "severe acute respiratory syndrome coronavirus 2" OR "novel coronavirus disease" OR "novel corona virus disease" OR "corona virus disease 2019" OR "coronavirus disease 2019" OR "novel coronavirus pneumonia" OR "novel corona virus pneumonia")  **2.** TI= (Azvudine OR FNC OR 2′-deoxy-2′-β-fluoro-4′-azidocytidine OR RO-0622 OR RO 0622 OR RO0622)  **3.** #1 AND #2 |
