# Supplementary material for: Effectiveness and safety of azvudine in COVID-19: A systematic review and meta-analysis
Source: PLoS One. 2024 Jun 13;19(6):e0298772. doi: 10.1371/journal.pone.0298772 (PMC11175417; doi:10.1371/journal.pone.0298772)
Supplement: S3 Table — (DOCX) [file pone.0298772.s008.docx]

S1 Table 2 - Risk of bias of included randomized controlled trial (RCT)

| Study | Random sequence generation | Allocation concealment | Blinding of participants and personnel | Blinding of outcome assessment | Incomplete outcome data | Selective reporting |
| --- | --- | --- | --- | --- | --- | --- |
| Ren 2020 | High | High | High | High | Low | Low |
| Silva 2023 | Low | Unclear | Low | Low | Low | Low |
| Souza 2023 | Low | Unclear | Low | Low | Low | Low |
