# Supplementary material for: Effectiveness and safety of azvudine in COVID-19: A systematic review and meta-analysis
Source: PLoS One. 2024 Jun 13;19(6):e0298772. doi: 10.1371/journal.pone.0298772 (PMC11175417; doi:10.1371/journal.pone.0298772)
Supplement: S4 Table — (DOCX) [file pone.0298772.s009.docx]

S1 Table 3 - ROBINS-I tool results for non-randomized studies

| Study | Confounding | Selection | Classification of interventions | deviations from intended interventions | Missing Data | Measurement  of outcomes | Reported  Result | Overall |
| --- | --- | --- | --- | --- | --- | --- | --- | --- |
| Chen 2023 | Moderate | Moderate | Low | Moderate | Low | Moderate | Low | Moderate |
| Deng 2022 | Low | Low | Low | Moderate | Low | Moderate | Low | Moderate |
| Dian 2023 | Low | Low | Low | Moderate | Low | Moderate | Low | Moderate |
| Fu 2023 | Moderate | Moderate | Low | Moderate | Low | Moderate | Low | Moderate |
| Gao 2023 | Low | Low | Low | Moderate | Low | Moderate | Low | Moderate |
| Han 2023 | Low | Low | Low | Moderate | Low | Moderate | Low | Moderate |
| Liu 2023 | Low | Low | Low | Moderate | Low | Moderate | Low | Moderate |
| Qi 2023 | Serious | Moderate | Low | Moderate | Low | Moderate | Low | Moderate |
| Qinqin Zhao 2023 | Moderate | Moderate | Low | Moderate | Low | Moderate | Low | Moderate |
| Shang 2023 | Low | Low | Low | Moderate | Low | Moderate | Low | Moderate |
| Shao 2023 | Moderate | Moderate | Low | Moderate | Low | Moderate | Low | Moderate |
| Shen 2023 | Low | Low | Low | Moderate | Low | Moderate | Low | Moderate |
| Sun 2023 | Low | Low | Low | Moderate | Low | Moderate | Low | Moderate |
| Wei 2023 | Low | Low | Low | Moderate | Low | Moderate | Low | Moderate |
| Xiang Zhao 2023 | Low | Low | Low | Moderate | Low | Moderate | Low | Moderate |
| Yang 2023 | Low | Low | Low | Moderate | Low | Moderate | Low | Moderate |
| Yiling Zhou 2023 | Moderate | Moderate | Low | Moderate | Low | Moderate | Low | Moderate |
| Zong 2023 | Low | Low | Low | Moderate | Low | Moderate | Low | Moderate |

Note: Moderate= the study is sound for a non-randomized study with regard to this domain but cannot be considered comparable to a well-performed randomized trial; Low=the study is comparable to a well-performed randomized trial with regard to this domain; Serious risk of bias =the study has some important problems
