# Supplementary material for: Effectiveness and safety of azvudine in COVID-19: A systematic review and meta-analysis
Source: PLoS One. 2024 Jun 13;19(6):e0298772. doi: 10.1371/journal.pone.0298772 (PMC11175417; doi:10.1371/journal.pone.0298772)
Supplement: S5 Table — (DOCX) [file pone.0298772.s010.docx]

S1 Table 4- Assessment of certainty of evidence using the GRADE approach for included outcomes

GRADE

| Certainty assessment | | | | | | | Effect | Certainty |
| --- | --- | --- | --- | --- | --- | --- | --- | --- |
| No. of studies | Study design | Risk of bias | Inconsistency | Indirectness | Imprecision | Other | Point estimate  (95% CI) |  |
| Mortality rate (AZ vs. SOC/PBO) | | | | | | | | |
| 9 | RCT, RCS | Serious | Not serious | Not serious | Not serious | None | RR= 0.48 (0.40 to 0.57) | Moderate |
| Mortality rate (AZ vs. NMV/r) | | | | | | | | |
| 7 | RCS | Serious | Not serious | Not serious | Not serious | None | RR= 0.73 (0.58 to 0.92) | Moderate |
| Negative PCR conversion time (AZ vs. SOC/PBO) | | | | | | | | |
| 4 | RCT, RCS | Serious | Very serious | Not serious | Not serious | None | SMD = - 0.75 (-1.29, - 0.21) | Low |
| Negative PCR conversion time (AZ vs. NMV/r) | | | | | | | | |
| 3 | RCS | Serious | Very serious | Not serious | Serious | None | SMD = 2.14 (-1.08 to 5.36) | Low |
| Length of hospital stay (AZ vs. SOC/PBO) | | | | | | | | |
| 4 | RCS | Serious | Very serious | Not serious | Serious | None | SMD = - 1.34 (-2.68 to 0.005) | Low |
| Length of hospital stay (AZ vs. NMV/r) | | | | | | | | |
| 3 | RCS | Serious | Very serious | Not serious | Not serious | None | SMD = - 0.49 (-1.52 to 0.52) | Low |
| ICU admission (AZ vs. SOC/PBO) | | | | | | | | |
| 3 | RCT, RCS | Serious | Not serious | Not serious | Not serious | None | RR= 0.68 (0.23 to 2.03) | Moderate |
| ICU admission (AZ vs. NMV/r) | | | | | | | | |
| 4 | RCS | Serious | Not serious | Not serious | Not serious | None | RR= 0.41 (0.21 to 0.78) | Moderate |
| Need for mechanical ventilation (AZ vs. SOC/PBO) | | | | | | | | |
| 4 | RCS | Serious | Not serious | Not serious | Not serious | None | RR= 0.90( 0.54 to 1.50) | Moderate |
| Need for mechanical ventilation (AZ vs. NMV/r) | | | | | | | | |
| 5 | RCS | Serious | Not serious | Not serious | Not serious | None | RR= 0.67( 0.51 to 0.89) | Moderate |
| Adverse events (AZ vs. SOC/PBO) | | | | | | | | |
| 7 | RCT, RCS | Serious | Very serious | Not serious | Not serious | None | RR= 1.03( 0.64 to 1.67) | Low |
| Adverse events (AZ vs. NMV/r) | | | | | | | | |
| 3 | RCS | Serious | Serious | Not serious | Not serious | None | RR= 0.60 (0.32 to 1.13) | Low |
